# Ruptured Hemorrhagic Ectopic Pregnancy Implanted in the Diaphragm: A Rare Case Report and Brief Literature Review

**DOI:** 10.3390/diagnostics11122342

**Published:** 2021-12-13

**Authors:** Ok Ju Kang, Ji Hye Koh, Ji Eun Yoo, So Yeon Park, Jeong-Ik Park, Songsoo Yang, Sang-Hun Lee, Soo-Jeong Lee, Jun-Woo Ahn, Hyun-Jin Roh, Jeong Sook Kim

**Affiliations:** 1 Department of Obstetrics and Gynecology, University of Ulsan College of Medicine, Asan Medical Center, Seoul 05505, Korea; lalala1301@gmail.com; 2 Department of Obstetrics and Gynecology, Ulsan University Hospital, University of Ulsan College of Medicine, Ulsan 44033, Korea; 0735445@uuh.ulsan.kr (J.H.K.); eunny501@gmail.com (J.E.Y.); synprk1001@naver.com (S.Y.P.); shlee73@uuh.ulsan.kr (S.-H.L.); exsjlee@uuh.ulsan.kr (S.-J.L.); ahnjwoo@uuh.ulsan.kr (J.-W.A.); 0729345@uuh.ulsan.kr (H.-J.R.); 3 Department of Surgery, Ulsan University Hospital, University of Ulsan College of Medicine, Ulsan 44033, Korea; jipark@uuh.ulsan.kr (J.-I.P.); ssyang@uuh.ulsan.kr (S.Y.)

**Keywords:** ectopic pregnancy, abdominal pregnancy, diaphragmatic pregnancy

## Abstract

The mortality and morbidity rates of non-tubal ectopic pregnancies with abdominal hemorrhaging are 7–8 times higher than those of tubal pregnancies. Diaphragmatic pregnancy is a rare non-tubal ectopic form, causing acute abdominal hemoperitoneum. Here, we present a case of a primary diaphragmatic ectopic pregnancy with hemorrhage that was immediately diagnosed and successfully managed with laparoscopic surgery. Rapid and accurate diagnosis using appropriate imaging modalities is critical for improving the prognosis of a child-bearing woman with an abdominal pregnancy.

## 1. Introduction

Ectopic pregnancy is the implantation of an embryo outside the uterine cavity. The estimated incidence of this abnormal pregnancy is 19.7 per 1000 pregnancies [1]. While most ectopic pregnancies (95.5%) occur in the fallopian tube, 1.3% arise in abdominal locations [2]. Extra-tubal pregnancies result from the direct implantation of the fertilized ovum onto the peritoneal surface or abdominopelvic organs such as the bowel, omentum, spleen, or liver [3]. These pregnancies are associated with high mortality and morbidity rates due to diagnostic difficulties [4,5]. Of the many implantation sites of ectopic pregnancy, only a few cases of diaphragmatic pregnancy have been reported.

Here, we present an unusual case of a primary diaphragmatic pregnancy causing acute abdominal hemoperitoneum.

## 2. Case Presentation

**Figure 1:**
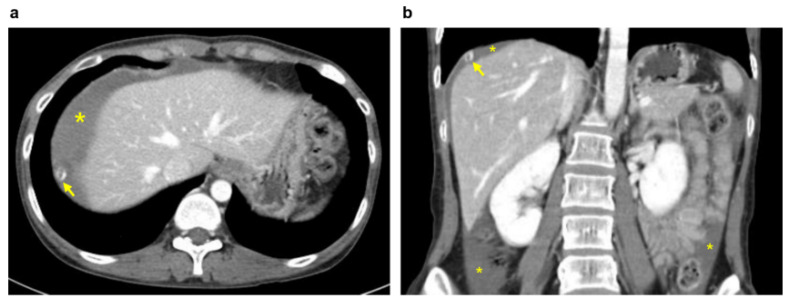
Abdominopelvic computed tomography images: A 2 cm target-like nodule with a thin, enhancing capsule (arrow); hemoperitoneum (*) in the axial (**a**) and coronal (**b**) planes.

**Figure 2:**
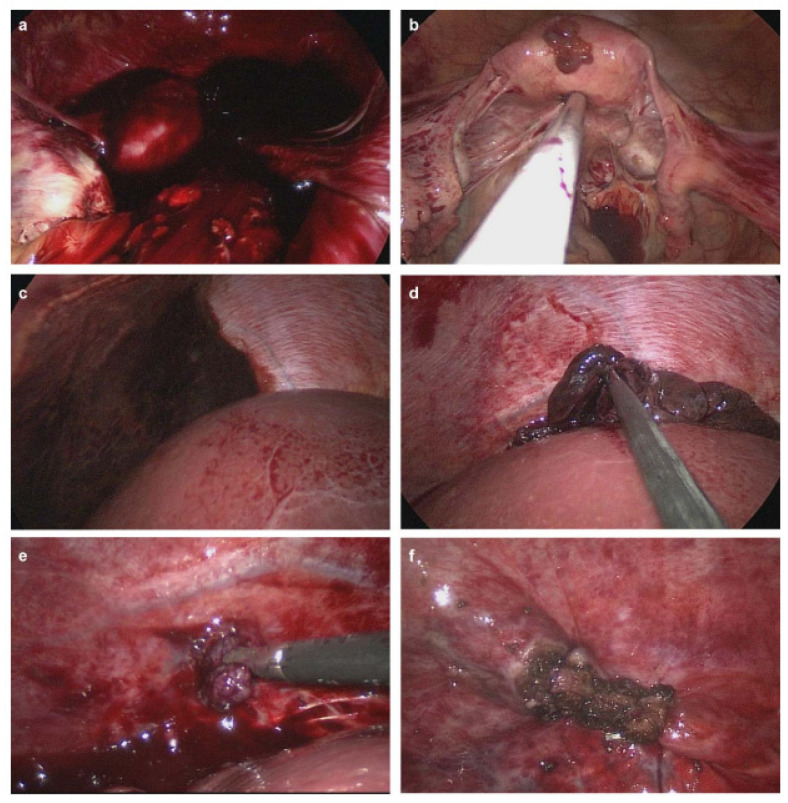
Images of laparoscopic surgery. (**a**) Blood clots in the pelvic cavity. (**b**) Normal uterus and bilateral tubes and ovaries. (**c**) Placental tissues with hematoma covering the diaphragm. (**d**) Removal of the placental tissues and hematoma. (**e**) Ectopic mass attached to, and invading, the diaphragm. (**f**) Complete removal of the ectopic mass from the diaphragm and suturing around the removal site.

**Figure 3:**
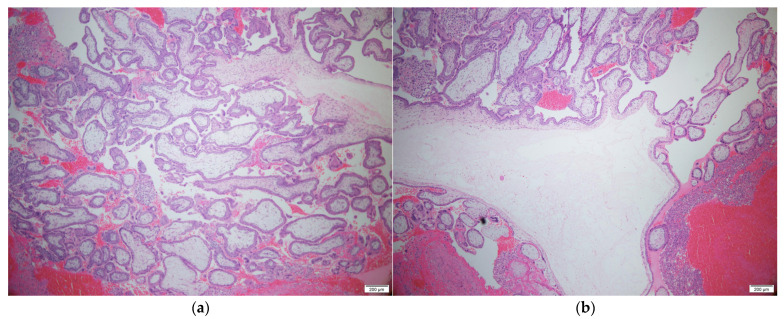
Histopathologic findings: Multiple chorionic villi in the periphery (**a**) and center (**b**) of the diaphragmatic ectopic disc are noted on H&E staining. (×50). Scale bar: 200µm.

A 34-year-old woman (gravida 3, para 3) with three spontaneous vaginal deliveries was transferred to the Ulsan University Hospital from a local clinic due to severe abdominal pain accompanied by right flank pain. The patient had been previously healthy and had no specific medical or surgical history. She had an irregular menstruation cycle, and her last menstruation occurred five weeks and six days previously. The initial vital signs at the emergency room were stable; systolic and diastolic blood pressure were 114 mmHg and 68 mmHg, respectively. The initial pulse rate was 71 beats per minute. Whole abdominal tenderness with muscle guarding was noted on physical examination. Blood tests showed a low hemoglobin level (10.7 g/dL). A urinary pregnancy test was positive, and the serum β-HCG level was 7377.0 mIU/mL. Gynecological sonography found no evidence of an intrauterine pregnancy, except for normal bilateral adnexa with free fluid collection, suggestive of hemoperitoneum. After eight hours, the follow up blood test showed a lower hemoglobin level (8.6 g/dL). Two packs of packed red blood cells were transfused. We suspected a ruptured ectopic pregnancy through elevated serum β-HCG, but the ectopic mass could not be identified on pelvic ultrasound. Thus, we planned abdominopelvic computed tomography (APCT) to determine the cause of the right frank pain. Approximately 2 cm hypervascular mass in the subphrenic region, with a moderate amount of hemoperitoneum, was revealed (Figure 1), which was thought to be the cause of the bleeding. Because of suspicions of a diaphragmatic ectopic pregnancy or other ruptured unknown hepatic mass, she was admitted for emergency surgery. Diagnostic laparoscopic surgery was performed in collaboration with a hepatobiliary surgeon and an obstetrician-gynecologist. On laparoscopy, about 400 mL of blood and clots were aspirated from the pelvic cavity, but both adnexa appeared normal. Approximately 20 × 10 cm tissue, suspected to be the placenta with a hematoma, had covered the diaphragm. After the removal of the placenta-like tissues, we found a 2 cm hypervascular mass attached to the diaphragm (Figure 2). The mass was completely resected from the diaphragm and sent for histological examination. After the surgical mass removal, the patient was discharged without any postoperative complications, and the serum β-HCG level normalized within a month. The final pathologic diagnosis indicated that the mass was a product of conception, consistent with an ectopic pregnancy (Figure 3).

## 3. Discussion

Abdominal ectopic pregnancies involving implantation of an embryo in the peritoneal cavity are rare, with an estimated incidence of approximately 1/10,000 pregnancies and 1/100 ectopic pregnancies [6,7]. Although these account for a small proportion of ectopic pregnancies, they are of clinical significance as the morbidity and mortality rates are 7–8 times higher than those of tubal pregnancies [8]. While the estimated maternal mortality rate for all ectopic pregnancies is reported as being between 2 and 4/1000 [9], the known maternal mortality rate associated with abdominal pregnancies is at least 5/1000 pregnancies, the highest of all types of ectopic pregnancies [7].

The classical triad of ectopic pregnancy consists of amenorrhea, vaginal bleeding, and abdominal pain. However, abdominal pregnancies remain associated with variable clinical symptoms because of the wide range of possible locations. Because of the rare incidence and complex clinical presentation, the diagnosis of abdominal pregnancies is often delayed or even missed preoperatively. Previous studies have described the diagnostic rate of abdominal pregnancy as 50–90% [10,11]. Advances in imaging modalities and improved sensitivity of urine and serum β-HCG tests have allowed for better diagnosis. A recent publication reported a successful diagnosis of a diaphragmatic ectopic pregnancy via CT, coupled with the detection of an elevated serum β-HCG level, on the first day of admission [12].

Depending on the fertilization site of the ovum, abdominal pregnancies are classified as either primary or secondary. A primary abdominal pregnancy refers to fertilization within the peritoneal cavity. If the tube ruptures and the fertilized egg implants elsewhere, it is classified as a secondary abdominal pregnancy. Primary abdominal pregnancies, which are very rare compared to secondary pregnancies, usually occur in the posterior wall of the uterus and the pouches of Douglas [7]. However, non-uterine implantations in the liver, spleen, or omentum have been reported [1,13,14]. In the present case, a primary abdominal pregnancy implanted in the diaphragm was confirmed according to Studdiford’s criteria [15]: (1) grossly normal fallopian tubes and ovaries, (2) the absence of uteroplacental fistula, (3) pregnancy exclusively related to the peritoneal surface, and (4) early diagnosis to eliminate the possibility of secondary implantation following primary implantation in the tube.

Although surgical management has been the preferred treatment [16,17], the standard management of abdominal pregnancies has not been established. Laparoscopic surgery is attempted first in early abdominal pregnancies [18,19]. It may, however, be difficult to detect and remove ectopic masses of abdominal pregnancies located in the upper abdomen, such as the spleen or liver. The conversion rate to laparotomy is approximately 27% [20]. In contrast, open laparotomy is required for advanced or complicated abdominal pregnancies because it allows for better access in managing the ectopic placenta and bleeding control [21]. Ectopic placentas should be removed because they can lead to severe complications such as hemorrhage, abscess, sepsis, and intestinal obstructions [22]. Additionally, arterial embolization of the placental site or systemic methotrexate (MTX) treatment is helpful for patients with an in situ placenta [23,24].

Medical therapy with MTX is also an option for unruptured abdominal pregnancies [1,25]. This treatment is recommended for symptom-free patients with a β-HCG concentration of less than 5000 IU/L. It is usually administered via systemic application or local injection, guided by either ultrasound (US) or hysteroscopy [26,27,28]. A recent case reported that US-guided microwave ablation may be an alternative treatment for ectopic pregnancies of the diaphragm [29]. A recent study showed that expectant management of tubal ectopic pregnancy was having higher clinical pregnancy (65.3%) and a feasible procedure within cut-off of serum β-HCG level 1745 mUI/mL and ectopic pregnancy mass size cut-off of 25 mm or smaller [30]. In surgical management for tubal pregnancy, it showed intrauterine pregnancy rates were similar (about 60%), but the two-year recurrent ectopic pregnancy rates were 5.3% for salpingectomy vs. 18.7% for salpingotomy. Persistent trophoblastic disease rates were 1.8% for salpingectomy vs. 12% for salpingotomy [31], but there was another study that showed no difference in reproductive outcomes among the different surgical techniques (salpingectomy, salpingostomy, and tubal milking) [32].

In our case, the diaphragmatic pregnancy was rapidly diagnosed through serum β-HCG testing and APCT as soon as the patient visited the emergency room. The ectopic mass was well-exposed and successfully removed through collaborative laparoscopic surgery. A ruptured ectopic pregnancy must always be considered as a differential diagnosis for childbearing women presenting with acute abdominal hemoperitoneum, even if the patient does not show the typical symptoms of an ectopic pregnancy. Moreover, if in doubt, the use of imaging modalities such as CT or magnetic resonance imaging is necessary for an accurate diagnosis and for providing prompt and appropriate treatment.

## Data Availability

Data are available on request.
